# Research on the expression of integrin β3 and leukaemia inhibitory factor in the decidua of women with cesarean scar pregnancy

**DOI:** 10.1186/s12884-017-1270-3

**Published:** 2017-03-11

**Authors:** Zhi-Da Qian, Yue Weng, Chun-Fen Wang, Li-Li Huang, Xiao-Ming Zhu

**Affiliations:** 1 Women’s Hospital, School of Medicine, Zhejiang University, 1 Xueshi Road, Hangzhou, Zhejiang Province 310006 People’s Republic of China; 2 Maternal and Child Health Institute of Lin’an City, 25 Jiangnan Road, Lin’an, Zhejiang Province 311300 People’s Republic of China; 3 Key Laboratory of Reproductive Genetics (Hangzhou, Zhejiang University), Ministry of Education, Hangzhou, People’s Republic of China

**Keywords:** Cesarean scar pregnancy, Decidua, Integrin, Leukaemia-inhibitory factor, Endometrial receptivity

## Abstract

**Background:**

Cesarean scar pregnancy (CSP) is a late serious complication of cesarean section. There has been an increase in the incidence of CSP worldwide in recent years. It’s a life-threatening condition because of the high risk of uncontrolled hemorrhage and uterine rupture. The mechanism of CSP is still unclear. The endometrial receptivity might be different in the cesarean scar between CSP and normal pregnancies. Endometrial expression of integrin β3 and LIF positively correlates with endometrial receptivity and embryo implantation. The purpose of the study is to explore the mechanism of CSP.

**Methods:**

The EnVision two-step immunohistochemical staining technique was used to detect the expression of integrin β3 and LIF in the decidua of women with CSP (20 cases) and normal pregnancies (20 cases). The distribution and staining intensity of integrin β3 and LIF in the two groups were observed. Observation of the staining were done using microscope within five randomly selected high-power fields (HPF, 10 × 40). All data analyses were conducted with SPSS 17.0 and the statistical significance was set at *P* <0.05.

**Results:**

The decidua in the different parts of both two groups that stained with the anti-integrin β3 and anti-LIF antibody: most of the integrin β3 and LIF positive cells were located in glandular epithelium. The expression intensity of integrin β3 in the cesarean scar in CSP group was significant higher than the uterine cavity in CSP group and the cesarean scar in normal pregnancy group. It’s similar with the uterine cavity in normal pregnancy group. The expression intensity of LIF in the cesarean scar in CSP group was significant higher than the uterine cavity in CSP group and the cesarean scar in normal pregnancy group. It’s significant lower than the uterine cavity in normal pregnancy group.

**Conclusions:**

The decidual integrin β3 and LIF might play an important role in the mechanism of CSP. The increase expression of integrin β3 and LIF in the cesarean scar decidua might be associated with embryo implantation in cesarean scar. The occurrence of CSP might be related to the changes of endometrial receptivity in local cesarean scar.

## Background

A cesarean scar pregnancy (CSP) is defined as a gestational sac located in the scar of a previous cesarean section (CS). It is a late serious complication of CS. The incidence of CSP is between 1:2226 and 1:1800 [1, 2]. CSP is rare. Over the past few years, with the rising rate of CS all over the world, there has been an increase in the incidence of CSP worldwide, especially in China [3]. CSP is considered a life-threatening condition because of the high risk of uncontrolled hemorrhage and uterine rupture, which might lead to a hysterectomy, with catastrophic consequences for the patient's reproductive future [4].

With limited experience and research of CSP, there is no consensus regarding the mechanism of CSP. The pathogenesis of CSP has not been clarified exactly yet. Embryo implantation is an important step of the reproductive process, and is critical to CSP. CSP is associated with embryo implantation in abnormal position (cesarean scar). Abnormalities in molecular factors of implantation and many other factors could be correlated to CSP. The most probable mechanism through which this can occur is invasion of the myometrium through a microscopic tract [5]. The tract is believed to develop from trauma from previous uterine surgeries like CS, dilatation and curettage and myomectomy. According to an earlier report, scar dehiscence was observed in 6%-10% of the women with a history of CS, which is a much higher rate than the incidence of CSP [6]. Cases have been reported of a healthy pregnancy occurring after CSP [7], and disruption of a cesarean scar does not necessarily lead to the development of repeated CSP.

Previous studies have shown that endometrial expression of integrin β3 subunit and LIF positively correlates with endometrial receptivity and embryo implantation [8]. Both integrin β3 subunit and LIF appeared in the endometrium coinciding with the window of implantation (WOI) and might be used as markers of endometrial receptivity.

The integrin β3 acts as an adhesion promoter via cell-cell interactions and it has been very well characterized within the human endometrium [9]. It is expressed in the glandular epithelium during the WOI, if pregnancy occurs [10]. Expression of integrin β3 is a biological marker for good endometrial receptivity and it plays a crucial role in blastocyst implantation [11]. Reduced expression of integrinβ3 has been related to unexplained infertility. Chen et al. [12] found that integrin β3 participated in ectopic implantation and might play a key role in tubal pregnancy.

LIF is a pleiotropic cytokine of the interleukin-6 family, which might play an important role in human reproduction [13]. It was initially identified by its ability to induce the macrophage differentiation of the myeloid leukaemic cell line M1 [14]. As one of the most important cytokines to influence endometrial receptivity during the early implantation window, LIF is involved in the regulation of implantation. In the absence of LIF, the mouse embryo can develop normally only to the blastocyst stage [15]. LIF plays a critical role in implantation in women [16]. Previous study showed that endometrial cell cultures from infertile women with repeated abortions or unexplained primary infertility were significantly lower when compared to fertile women [17].

LIF might contribute to the development of ectopic pregnancies. Immunohistochemical labeling of LIF in the fallopian tube was found to be increased in ectopic pregnancies compared to non-pregnant and healthy pregnant controls. This might indicate a role of LIF in the ectopic implantation of embryos [18]. LIF might facilitate the development of ectopic pregnancy by stimulating blastocyst adhesion and trophoblast outgrowth from placental explants [19].

Integrin β3 and LIF are the two cellular factors which have been largely accepted as the promising candidates of biomarkers of uterine receptivity in human. They are known to relate to tubal pregnancy. However, It is not clear whether the blastocyst requires expression of integrin β3 or LIF for implantation into cesarean scar other than cavity endometrium; such as in CSP. They might be used to evaluate the change of endometrial receptivity in cesarean scar in CSP patients.

We assumed that: The endometrial receptivity might be different in the cesarean scar between CSP and normal early intrauterine pregnancies. The change of local endometrial receptivity in the cesarean scar might be beneficial to the embryo implantation in CSP patients. We carried out an immunohistochemical research on the expression of decidual integrin β3 and LIF in women with CSP. This study aimed to: (a) test whether integrin β3 and LIF are localized in cesarean scar decidua in CSP patients; (b) investigate whether decidual integrin β3 and LIF in CSP patients are different from that of healthy controls; and (c) explore the change of endometrial receptivity in the mechanism of CSP.

## Methods

### The diagnosis standard of CSP

**Fig. 1:**
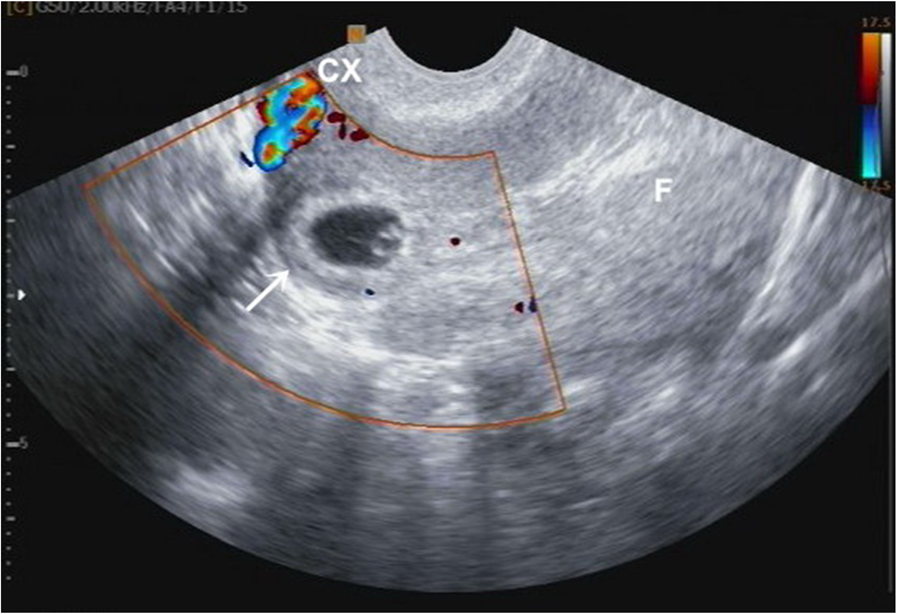
A cesarean scar pregnancy demonstrated by ultrasound showing the gestational sac implanted in the lower segment dehiscence of the anterior myometrium. The residual myometrium was thin and the amniotic sac bulged into the uterovesical fold under the cesarean scar (indicated by the arrow). CX: cervix; F: fundal endometrial cavity

The CSP was diagnosed using the following criteria: a history of CS in the lower uterine segment, positive serum beta-human chorionic gonadotropin (β-HCG) level, and fulfillment of the following ultrasonography standard [20] (Fig. 1): (a) an empty uterine cavity and cervical canal; (b) development of the gestational sac in the anterior portion of the lower uterine segment; and (c) absence of healthy myometrium between the bladder and the gestational sac.

### Subjects

This is an immunohistochemical study of integrin β3 and LIF in the uterine decidua. The specimens (the decidua from cesarean scar and uterine cavity) were obtained from the Department of Obstetrics and Gynecology, Women’s Hospital, School of Medicine, Zhejiang University, China, between October 2013 and September 2014. This hospital is the largest obstetrics and gynecology medical treatment center in Zhejiang Province. Samples of CSP group (group A, 20 cases) were collected from women with CSP by ultrasound (LOGIQ P6, GE, USA) guided curettage. Samples of the normal pregnancy group (group B, 20 cases) were collected from women who underwent termination of normal early pregnancies with CS history in the same hospital. Each case (both CSP and normal pregnancy with cesarean scar patients) contains two samples of decidua in the different position: the cesarean scar and the uterine cavity. All women were from 20 to 40 years of age with gestation of less than 10 weeks. Exclusion criteria included inevitable abortion, missed abortion, incomplete abortion, cervical pregnancy, genetic, endocrine and immune disorders, cancer, genital anatomical abnormalities and major surgical diseases, history of long-term medication, trophoblastic disease, those undergoing endocrine therapy, chemotherapy, and radiotherapy, and significant maternal cardiac, renal, hepatic, and blood system disease. Written informed consent for the surgery and participation was obtained from each patient in the study, and the protocol was approved by the Institutional Review Board in Women’s Hospital, School of Medicine, Zhejiang University.

### Immunohistochemistry

Decidual samples were immediately placed into a 10% formaldehyde solution bag and fixed for 10 to 24 h. Paraffin blocks were sectioned into a 4 μm thickness. The EnVision two-step immunohistochemical staining technique was used to detect the expression of integrin β3 and LIF in the decidua in the different parts. The immunohistochemical staining was done by one person only. Positive controls were included in every batch of tests. Primary antibodies: Rabbit antihuman integrin β3 (clone number EPR2417Y) was obtained from Abcam Biotechnology Co., Ltd, USA. Mouse antihuman LIF (clone number IMG39N7D10) was obtained from Abcam Biotechnology Co., Ltd, USA. The secondary antibody used was ChemMatei Envisioni Detection Kit Peroxidase/DAB, from DAKO Biotechnology Co., Ltd, Denmark. The human normal tonsil tissue was used as an integrin β3 positive control, and the mouse colon tissue was used as a LIF positive control.

Positive cells displayed brown membrane and cytoplasmic staining features. The distribution and quantity of two markers in the two groups were observed. Semi-quantitative analysis was done by staining intensity observation using microscope within five randomly selected high-power fields (HPF, 10 × 40). All the immunostained slides were reviewed by two observers independently. Observation of the staining was done using an Olympus CHK microscope (Olympus, Japan). Staining intensity of tissue sections was evaluated and graded (0, absence; 1, weak; 2, moderate; 3, strong). It was assessed by calculating the total score of five HPF. Photomicrographs were taken using a digital camera (Nikon, Japan).

### Statistics

The general information of the two groups was evaluated. The difference in the expression intensity of decidual integrin β3 and LIF in the cesarean scar and uterine cavity between the two groups was evaluated. All data analyses were conducted with SPSS 17.0 (SPSS Inc., USA). A two-tailed significance test was used for all comparisons, and statistical significance was set at *P* < 0.05. Data were analyzed for normal distribution with the Kolmogorov-Smirnov test and for homogeneity of variance with the Levene test. If variables met assumptions of normality and homogeneity of variance, an independent samples *t* test was used. If not, a nonparametric test (the Mann-Whitney *U* test) was used.

## Results

A total of 40 patients were enrolled in the study. Histopathologic examination performed on tissue obtained at surgery confirmed the diagnosis of CSP and normal early pregnancies. There were no significant differences of the baseline parameters of subjects in the two groups (Table 1).

**Table 1:** Patient characteristics and demographics

Characteristic	Group A (*n* = 20)	Group B (*n* = 20)	*P* value
Maternal age (years)	33.80 ± 4.70	31.50 ± 3.98	0.103^a^
Abortion (times)	2.05 ± 1.64	1.30 ± 1.03	0.091^a^
Gestational age (days)	45.00 ± 5.73	47.45 ± 7.12	0.238^a^
Gestational sac diameter (cm)	2.30 ± 0.84	2.27 ± 0.77	0.907^a^
Number of prior CS [times, median (range)]	1 (1–2)	1 (1–2)	0.602^b^
Interval between CS and CSP (months)	64.50 ± 53.27	50.35 ± 30.16	0.308^a^

Normal distribution (mean ± SD), non-normal distribution [median (range)]. Unless noted otherwise, values are presented as mean ± SD

*P* values for specific categories refer to differences present among the categories

*CS* cesarean section, *CSP* cesarean scar pregnancy

^a^ *P* value is from independent samples *t* test

^b^ *P* value is from Mann-Whitney test

### The location of decidual integrin β3

**Fig. 2:**
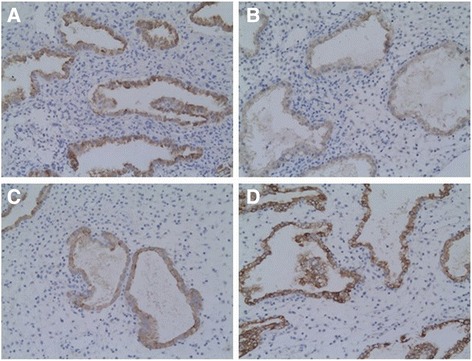
Representative pictures of immunohistochemical expression of decidual integrin β3 subunit in cesarean scar and uterine cavity in the two groups. **a** CSP-cesarean scar decidua (A strong positive immunohistochemical staining in glandular epithelial cells, 10 × 20); **b** CSP-uterine cavity decidua (A medium positive immunohistochemical staining in glandular epithelial cells, 10 × 20); **c** Normal early pregnancy-cesarean scar decidua (A medium positive immunohistochemical staining in glandular epithelial cells, 10 × 20); **d** Normal early pregnancy-uterine cavity decidua (A strong positive immunohistochemical staining in glandular epithelial cells, 10 × 20)

The decidua in the different parts of both two groups stained with the anti-integrin β3 antibody: most of the integrin β3 positive cells in the decidua from the two groups were located in maternal uterine epithelium and glandular epithelium (membrane and cytoplasm staining) and very few of these cells were present at very weak staining in the stroma (Fig. 2).

### The staining intensity of decidual integrin β3

The staining intensity of decidual integrin β3 in the cesarean scar in CSP group was 13.35 ± 1.18/5HPF: significant higher than the uterine cavity in CSP group (9.90 ± 2.10/5HPF, t = 6.402, *p* =0.000); significant higher than the cesarean scar in normal pregnancy group (8.10 ± 1.71/5HPF, t = 11.278, *p* =0.000); similar with the uterine cavity in normal pregnancy group (13.45 ± 1.05/5HPF, t = −0.283, *p* =0.779).

### The location of decidual LIF

**Fig. 3:**
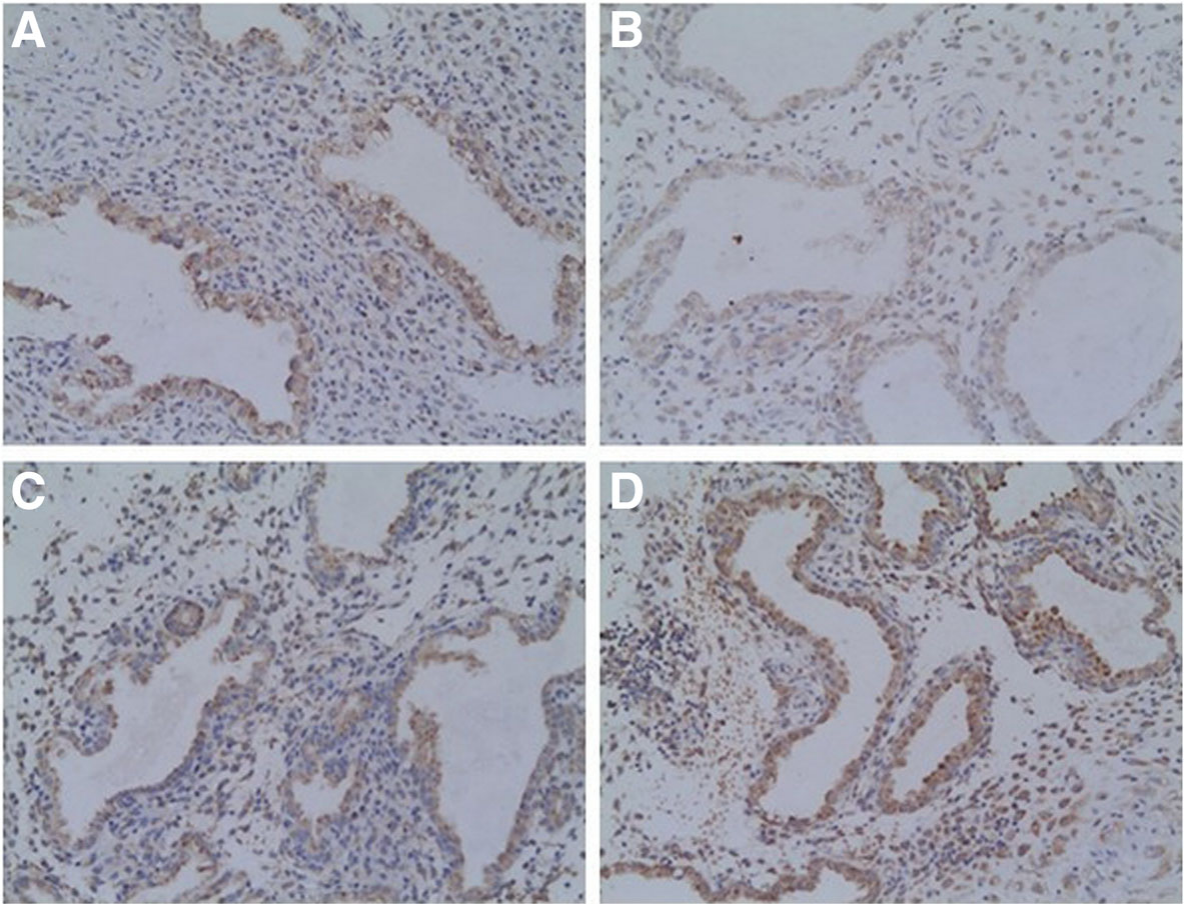
Representative pictures of immunohistochemical expression of LIF in cesarean scar decidua and uterine cavity decidua in the two groups. **a** CSP-cesarean scar decidua (A medium positive immunohistochemical staining, primarily in glandular epithelial cells, 10 × 20); **b** CSP-uterine cavity decidua (A weak positive immunohistochemical staining, primarily in glandular epithelial cells, 10 × 20); **c** Normal early pregnancy-cesarean scar decidua (A weak positive immunohistochemical staining, primarily in glandular epithelial cells, 10 × 20); **d** Normal early pregnancy-uterine cavity decidua (A medium positive immunohistochemical staining, primarily in glandular epithelial cells, 10 × 20)

The decidua in the different parts of both two groups was also positive for LIF: most of the LIF positive cells in the decidua from the two groups were located in maternal uterine epithelium and glandular epithelium (membrane and cytoplasm staining) and some of these cells were present at weak staining in the stroma (Fig. 3).

### The staining intensity of decidual LIF

The staining intensity of decidual LIF in the cesarean scar in CSP group was 7.75 ± 1.80/5HPF: significant higher than the uterine cavity in CSP group (5.25 ± 0.55/5HPF, t = 5.932, *p* =0.000); significant higher than the cesarean scar decidua in normal pregnancy group (6.65 ± 1.50/5HPF, t = 2.100, *p* =0.042); significant lower than the uterine cavity in normal pregnancy group (10.00 ± 1.97/5HPF, t = −3.746, *p* =0.001).

## Discussion

CSP is an infrequent type of ectopic pregnancy. It carries a high risk of uterine rupture and uncontrollable bleeding. It is important to maintain a high index of clinical suspicion. The etiology of cesarean scar implantation is still far from complete, but the bulk of the existing literature supports the hypothesis that it arises from a combination of cesarean scar disruption (the trauma theory), cytokines and enzymes changes in the cesarean scar environment. However, with limited research, the precise mechanism of CSP is unknown, factors critical for embryo implantation into the uterus might also contribute to blastocyst implantation in the cesarean scar.

For implantation to occur, the blastocyst must first lose the zona pellucida to allow intimate adhesion between the trophectoderm and the uterine epithelial surface. A WOI has also been proposed to occur in the cesarean scar, during which time the cesarean scar epithelium is susceptible to ectopic implantation. The change of local endometrial receptivity in the cesarean scar might be beneficial to the embryo implantation in CSP patients. Therefore, immunohistochemical labeling of integrin β3 and LIF in the cesarean scar and uterine cavity region from women with CSP and intrauterine pregnancy were studied in this research.

This investigation revealed that the decidua in the different parts of both two groups were positive for integrin β3. Most of the integrin β3 positive cells in the decidua were located in maternal uterine epithelium and glandular epithelium. The expression of integrin β3 showed statistically significant differences in different parts between the two groups. Integrin β3 expression in cesarean scar decidua was statistically significantly increased in women with CSP compared to normal early pregnant women. It demonstrates that the increase expression of integrin β3 in the cesarean scar decidua might be associated with embryo implantation in cesarean scar, which might lead to CSP.

Our results are similar with Chen et al. [12]. They found that expression of integrin β3 in tubal epithelium of tubal pregnancy was higher than that in normal tubal epithelium, but lower than that in normal decidual cells. Integrin β3 might play a role in ectopic pregnancy. A previous study revealed that high levels of integrin β3 expression correlate with the appearance of pinopodes on postovulatory days 7 to 8 [21], which provides a characteristic biomarker for endometrial receptivity during the early implantation window phase [10]. Other authors found that a reduction of integrin β3 during the implantation window in patients with recurrent pregnancy loss, compared to controls, either in frozen sections [22, 23].

Endometrium is composed of various cells and the extracellular matrix composition (ECM), containing abundant integrin. To implant, the blastocyst needs to aggressively adhere to the endometrium so that it can be provided with oxygen and nutrients. It is necessary to have a receptive endometrium that allows the invasion of the blastocyst and the rapid growth of the placenta while supporting the transformation of uterine into decidual cells. The important role of integrin in embryo implantation is gradually recognized. The integrins are a family of widely expressed heterodimeric cell surface receptors that mediate cell-cell and ECM adhesion. In addition to providing a physical transmembrane link between the extracellular environment and the cytoskeleton, they are capable of transducing bi-directional signals across the cell membrane [24]. ECM-integrin-cytoskeleton complex is the basic structure of integrin signaling pathway. Integrin-mediated signaling pathways play very important roles in embryo implantation, including focal adhesion kinase (FAK), protein kinase B (PKB) and extracellular signal-regulated protein kinase (ERK) [25, 26]. Kabir-Salmani et al. indicated that integrin β3 localization in the core of focal adhesions of extravillous trophoblast cells and that integrin β3 signaling pathways are activated in insulin-like growth factor-I (IGF-I)-mediated migration of these cells [27].

We consider that the maternal glandular epithelium and uterine epithelium are close to gestational sac (the maternal-fetal interface). Integrin β3 and other cytokines might be involved in gathering the local response in the cesarean scar decidua during the embryo implantation. The decidual integrin β3 might play an important role in the mechanism of CSP. CS causes the injury of myometrium and basal layer of the endometrium. The formation of scar tissue and fibrosis is in response to CS damage. There might be some postoperative complications that affect uterine incision healing. Then, a microscopic tract is believed to develop from the trauma of previous CS. After a CS, women could attempt another pregnancy. Receptivity of endometrium is influenced by many factors, and it is not benefit embryo implantation. Integrin on trophoblast is combined with ligand on cesarean scar endometrium, which facilitating embryo recognition and attachment. The embryo is ectopic implanted in the cesarean scar and eventually lead to the occurrence of CSP.

This investigation revealed that the decidua in the different parts of both two groups were positive for LIF. Most of the LIF positive cells in the decidua were located in maternal uterine epithelium and glandular epithelium. The expression of LIF showed statistically significant differences in different parts between the two groups. LIF expression in cesarean scar decidua was statistically significantly increased in women with CSP compared to normal early pregnant women. It suggests that the increase expression of LIF in the cesarean scar decidua might be associated with embryo implant in cesarean scar, which might lead to CSP.

A previous study revealed that LIF expression in the fallopian tube was found to be increased in ectopic pregnancies compared to non-pregnant and healthy pregnant controls [18]. Our results are similar with it. LIF might contribute to the development of ectopic pregnancies by facilitating the development of ectopic pregnancy by stimulating blastocyst adhesion and trophoblast outgrowth from placental explants [19].

LIF is essential for embryo implantation. LIF regulates trophoblast function and vascular formation in the placenta. It is essential for embryo implantation in the endometrium. The expression of LIF has been demonstrated in the uterus of a variety of mammals. In most species, endometrial LIF expression increases around the time of blastocyst implantation. The expression of LIF and its receptor (LIFR) in human endometrium reaches peak levels during the peri-implantation phase [28]. In many species, LIF remains raising throughout the postovulatory phase and this might reflect a need for this cytokine throughout prei-implantation blastocyst development. LIF expression is also elevated during early pregnancy in many species investigated.

LIF is a secreted glycoprotein that signals via the gp130/LIFR complex to activate the Janus tyrosine kinases (JAK). This, in turn, can activate downstream signaling pathways, including the signal transducer and activator of transcription (STAT) pathway [29], or extracellular signal regulated kinase (ERK) [30]. In the human endometrium and trophoblast, LIF primarily acts via STAT3 [31]. LIF is also involved in regulating the balance between type 1 T-helper (Th1) and type 2 T-helper (Th2), and the Th1/Th2 balance regulates the maintenance of the pregnancy [32]. A shift from Th1 to Th2 response at the maternal-fetal interface may contribute to successful pregnancy [33].

When women with a microscopic tract developped from previous CS attempt another pregnancy, LIFR on trophoblast is combined with LIF on cesarean scar endometrium, which facilitating embryo recognition and attachment. LIF might stimulate blastocyst adhesion and trophoblast outgrowth from placental explants. Then, the embryo is ectopic implanted in the cesarean scar and eventually lead to the occurrence of CSP.

There are a few weaknesses in this study. For example, some limitations of this study due to the small sample size and semi-quantitative evaluation observed. It is also possible that the different staining intensity of integrin β3 and LIF might represent a local response to the pregnancy, rather than a cause of ectopic pregnancy. Previous reports have verified a cycle-dependent expression of the integrin β3 and LIF in the human endometrium [10, 28]. The expression of decidual integrin β3 and LIF in early pregnancy stages might be different from WOI. However, CSP is a rare type of ectopic pregnancy. The occurrence of CSP is very difficult to predict. We could not obtain the specimens around the implantation window in CSP patients. So we collected and studied the decidua in early pregnancy stages, to speculate the changes of endometrial receptivity during the implantation window.

## Conclusions

The etiology of CSP remains unknown in spite of an increasing interest. In CSP, embryo implantation is an extremely complex physiological process regulated by many factors in the uterus and embryo, and the underlying mechanism has not yet been fully elucidated. Using immunohistochemical analysis, we found that integrin β3 subunit and LIF were expressed in the decidua of CSP patients and normal pregnant women. There were significant differences in the staining intensity of decidual integrin β3 subunit and LIF between CSP group and the control group during the early pregnancies. Our results indicated that: (a) the decidual integrin β3 and LIF might play an important role in the mechanism of CSP; (b) the increase expression of decidual integrin β3 and LIF in the cesarean scar might be associated with embryo implantation in cesarean scar, which might lead to a CSP; and (c) the occurrence of CSP might be related to the changes of endometrial receptivity in local cesarean scar. However, embryo implantation is affected by both the embryo and endometrium in CSP patients. Considering the rising incidence of CSP and its catastrophic consequences, further studies are needed. Despite awareness of the limitations of this study, the results encourage further larger studies in this promising research field.

## Abbreviations

CS: Cesarean section
CSP: Cesarean scar pregnancy
ECM: Extracellular matrix composition
ERK: Extracellular signal regulated protein kinase
FAK: Focal adhesion kinase
IGF-I: Insulin-like growth factor-I
JAK: Janus tyrosine kinases
LIF: Leukaemia inhibitory factor
LIFR: Leukaemia inhibitory factor receptor
PKB: Protein kinase B
STAT: Signal transducer and activator of transcription
Th1: Type 1T-helper
Th2: Type 2T-helper
WOI: Window of implantation
β-HCG: Beta-human chorionic gonadotropin
